# Supplementary figures and images for: Primary productivity as a control over soil microbial diversity along environmental gradients in a polar desert ecosystem
Source: PeerJ. 2017 Jul 25;5:e3377. doi: 10.7717/peerj.3377 (PMC5530992; doi:10.7717/peerj.3377)

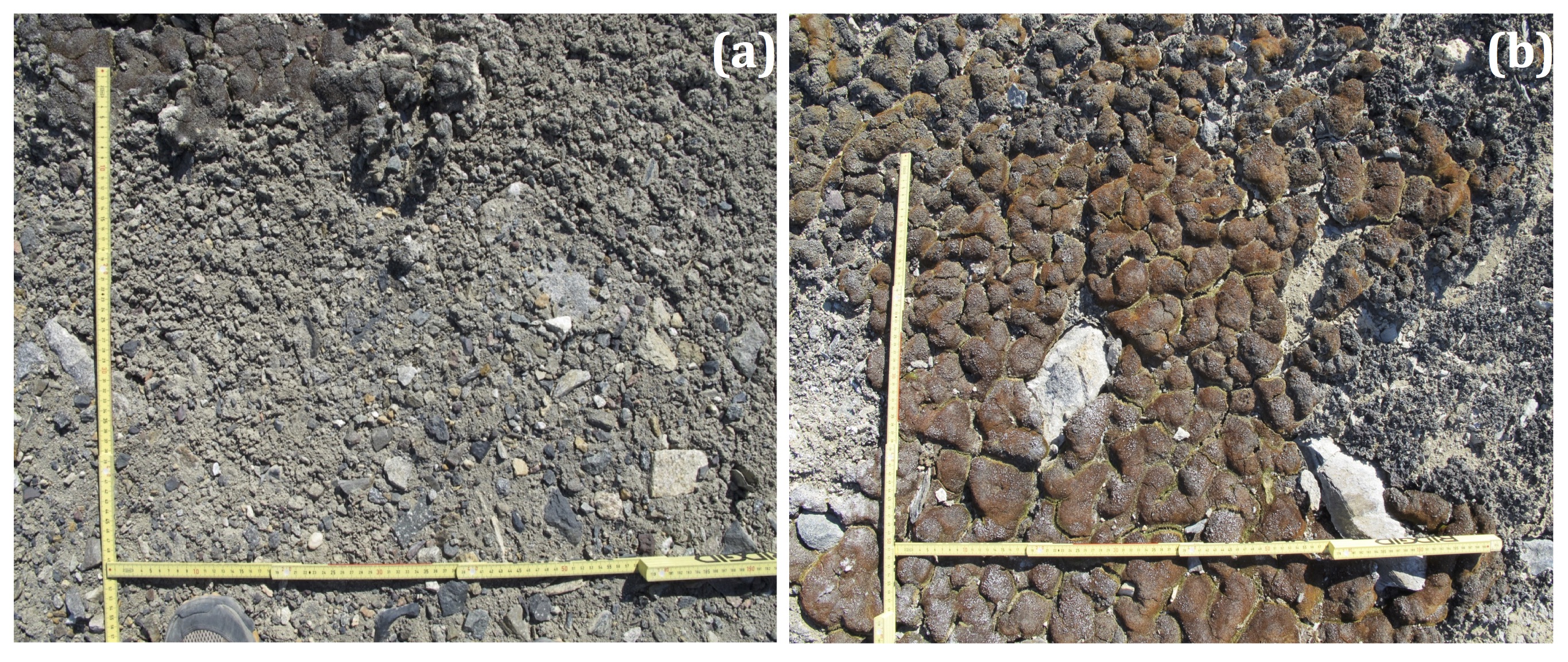

Supplement: Supplemental Information 1 — Example sampling locations from low (left) and high (right) productivity zones. Each location (50 cm × 50 cm) was sampled three times for surface cryptogams and underlying soils, while PAM fluorometry was used to collect estimates of electron transport rate from eight points along a gridded pattern. [file peerj-05-3377-s001.jpg]
